# Supplementary figures and images for: Eculizumab in patients with severe coronavirus disease 2019 (COVID-19) requiring continuous positive airway pressure ventilator support: Retrospective cohort study
Source: PLoS One. 2021 Dec 20;16(12):e0261113. doi: 10.1371/journal.pone.0261113 (PMC8687582; doi:10.1371/journal.pone.0261113)

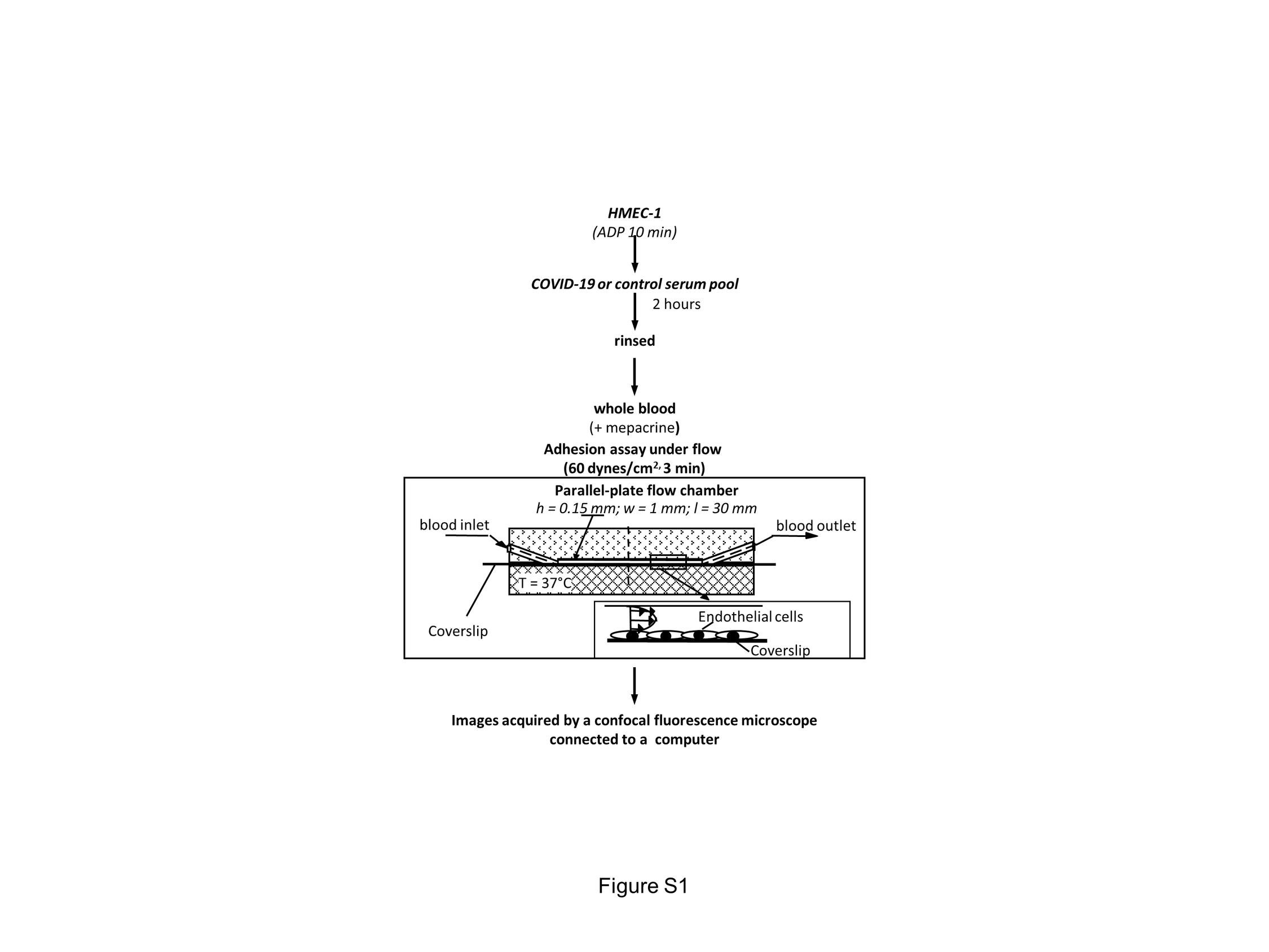

Supplement: S1 Fig — HMEC-1 were treated with ADP and exposed for 2 h in static conditions to 50% serum from COVID-19 patients, from aHUS patients studied in remission (aHUS positive controls) or from control serum pool diluted with test medium. Perfusion of heparinised whole blood from healthy subjects (added with mepacrine) was then performed in a thermostatic flow chamber in which one surface of the perfusion channel was a glass slide seeded with a monolayer of endothelial cells, at a constant flow rate of 1500 sec-1 (60 dynes/cm2). After 3 min, perfusion was stopped, and the slide with the endothelial cell monolayer was dehydrated and fixed. The values were expressed in pixels2. (TIF) [file pone.0261113.s001.tif]
