# Supplementary material for: Eculizumab in patients with severe coronavirus disease 2019 (COVID-19) requiring continuous positive airway pressure ventilator support: Retrospective cohort study
Source: PLoS One. 2021 Dec 20;16(12):e0261113. doi: 10.1371/journal.pone.0261113 (PMC8687582; doi:10.1371/journal.pone.0261113)
Supplement: S1 Table — (DOCX) [file pone.0261113.s002.docx]

**Table S1.** **Causes of death in the study group as a whole, in patients treated with eculizumab and in controls considered separately.**

|  | **Overall**  *(n=33)* | **Eculizumab**  *(n=2)* | **Controls**  *(n=31)* |
| --- | --- | --- | --- |
| Respiratory insufficiency | 23 (69.7) | 2 (100.0) | 21 (67.7) |
| Multiple organ failure | 4 (12.1) | 0 | 4 (12.9) |
| Septic shock | 3 (9·1) | 0 | 3 (9.7) |
| Cardiogenic shock | 2 (6.1) | 0 | 2 (6.4) |
| Myocardial infarction | 1 (3.0) | 0 | 1 (3.2) |

Data are numbers (percentages).
