# Supplementary material for: Eculizumab in patients with severe coronavirus disease 2019 (COVID-19) requiring continuous positive airway pressure ventilator support: Retrospective cohort study
Source: PLoS One. 2021 Dec 20;16(12):e0261113. doi: 10.1371/journal.pone.0261113 (PMC8687582; doi:10.1371/journal.pone.0261113)
Supplement: S2 Table — (DOCX) [file pone.0261113.s003.docx]

**Table S2. Age, gender and chronic complications at hospital discharge in patients treated with eculizumab and in controls.**

| **Study group** | **Age, *years*** | **Gender** | **Chronic condition at discharge** |
| --- | --- | --- | --- |
| Eculizumab | 55 | Male | Exertional dyspnea |
| Eculizumab | 49 | Male | Polyneuropathy (Guillain-Barré syndrome) with residual gait impairment |
| Control | 68 | Male | Paroxysmal atrial fibrillation |
| Control | 81 | Female | Asthenia and gait impairment |
| Control | 83 | Male | Sequelae of pulmonary thrombo-embolism |
| Control | 78 | Male | Asthenia, dyspnea |
| Control | 66 | Male | Exertional dyspnea |
| Control | 78 | Female | Sequelae of pulmonary thrombo-embolism |
| Control | 73 | Male | Atrioventricular block, asthenia |
| Control | 81 | Female | Paroxysmal supraventricular tachycardia |
| Control | 75 | Male | Sequelae of pulmonary thrombo-embolism |
| Control | 78 | Male | Cognitive deficits, asthenia |
| Control | 68 | Male | Sequelae of pulmonary thrombo-embolism |
| Control | 79 | Male | Hypokynetic syndrome |
| Control | 52 | Male | Motor deficits |
| Control | 61 | Male | Sequelae of pulmonary thrombo-embolism |
| Control | 60 | Male | Dyspnea |
| Control | 46 | Male | Exertional dyspnea, asthenia |
| Control | 57 | Male | Amnesia, polyneuropathy |
| Control | 68 | Female | Sequelae of pulmonary thrombo-embolism |
| Control | 86 | Female | Asthenia |
| Control | 70 | Male | Respiratory failure needing oxygen therapy |
| Control | 48 | Male | Exertional dyspnea, asthenia |
