# Supplementary material for: Eculizumab in patients with severe coronavirus disease 2019 (COVID-19) requiring continuous positive airway pressure ventilator support: Retrospective cohort study
Source: PLoS One. 2021 Dec 20;16(12):e0261113. doi: 10.1371/journal.pone.0261113 (PMC8687582; doi:10.1371/journal.pone.0261113)
Supplement: S1 Appendix — (DOCX) [file pone.0261113.s004.docx]

**S1 Appendix. Supplementary Methods**

**Study population**

Participants were more than 18-year-old subjects who were admitted at the COVID Unit of the Azienda Socio Sanitaria Territoriale (ASST) Papa Giovanni XXIII in Bergamo (Italy) because of severe respiratory distress due to COVID-19 and receiving CPAP ventilator support from 24 hours or less. The diagnosis of COVID-19 was based on the 19 March 2020 WHO Interim guidance criteria [1], including history of exposure along with typical symptoms and clinical, radiological and laboratory findings upon admission. Radiologic assessments and all laboratory tests, including arterial blood gas analysis, were performed according to local clinical practice and based on clinical needs. The diagnosis was confirmed by detection at admission of SARS-CoV-2 genome from nasal swabs and respiratory samples by using two different molecular methods (GeneFinder COVID-19-Elitech Group, Allplex™ 2019-nCoV Assay - Seegene Inc) according to the manufacturer’s instructions. After the purification of viral RNA from clinical samples, the detection of RdRp, E and N viral genes was obtained by real time Polymerase Chain Reaction (RT-PCR) according to WHO protocol [1,2].

Ten participants received eculizumab treatment in the context of the FDA approved program of off-label compassionate use of the anti-C5 monoclonal antibody for the treatment of non-intubated patients with COVID-19 (ClinicalTrials.gov Identifier: NCT04288713). The drug was freely supplied by the manufacturer (Alexion Pharma Italy S.R.L., Milan). All of them provided written informed consent to eculizumab off-label treatment. According to guidelines, before the first eculizumab administration patients received antibiotic coverage against capsulated bacteria including Neisseria Meningitis and Pneumococcus. No participant received compensation for eculizumab therapy. The compassionate treatment protocol was approved by the local Ethical Committee.

Eculizumab-treated patients and controls who received the same standard supportive therapy but no eculizumab, were admitted at the same COVID Unit and required CPAP ventilator support since 24 hour or less because of severe respiratory insufficiency. In controls the diagnosis of COVID-19 was based on the same WHO criteria considered to establish the diagnosis in the ten contemporary patients. The protocol of this controlled study was also approved by the local Ethical Committee.

**Eculizumab administration**

Before infusion, eculizumab was diluted to a final concentration of 5 mg/ml and the final solution was infused intravenously over approximately 35 minutes. An intensivist was alerted before any eculizumab administration and clinical symptoms and vital signs were monitored during the infusion and for at least one hour after its completion in order to immediately capture any potential infusion reaction.

**In vivo complement activity**

SC5b-9 levels were evaluated in plasma EDTA by MicroVue SC5b-9 Plus EIA (SC5b-9 Plus; Quidel).

**Ex vivo complement deposition and thrombi formation on HMEC-1**

Ex-vivo serum-induced C5b-9 deposits and thrombi formation were evaluated as described previously, with minor modifications [3–5].

**Complement Deposition *-*** Human microvascular endothelial cells of dermal origin (HMEC-1 cell line, a gift from Dr Edwin Ades and Francisco J. Candal of CDC and Dr Thomas Lawley of Emory University, Atlanta, GA) were plated on glass coverslips and used when confluent. Cells were activated with 10 µM ADP (Sigma) for 10 minutes and then incubated for 2 hours with serum from COVID-19 patients or aHUS patients, or from healthy controls (n=17) or with a pool of sera from 10 healthy subjects, diluted 1:2 with test medium (HBSS with 0.5% BSA).

Thereafter, HMEC-1 were fixed in 3% paraformaldehyde and stained with rabbit anti–human complement C5b-9 complex antibody (Calbiochem) followed by FITC-conjugated secondary antibody (Jackson Immuno Research Laboratories). An AXIO Imager.Z2 laser microscope was used for acquisition of the fluorescent staining on endothelial cell surface. Fifteen fields per sample were acquired and the area occupied by the fluorescent staining was evaluated by automatic edge detection using built-in specific functions of the software Image J and the highest and lowest values discarded. Results were expressed as percent of staining in respect to a control serum pool run in parallel.

**Thrombus formation** - HMEC-1 of dermal origin were plated on glass slides and used when confluent. Cells were activated with 10 µM ADP for 10 minutes, and then incubated for 2 hours with serum from patients or with a pool of sera from 10 healthy controls diluted 1:2 with test medium (HBSS with 0.5% BSA). In each experiment, the control serum pool was tested in parallel with patient serum. Thereafter, HMEC-1 were perfused in a flow chamber with heparinized whole blood (10 UI/ml) obtained from healthy subjects (added with the fluorescent dye mepacrine that labels platelets). After 3 min of perfusion, the endothelial cell monolayer was fixed in acetone. Fifteen images per sample of platelet thrombi on endothelial cell surface were acquired by confocal inverted laser microscope, and areas occupied by thrombi evaluated using Image J ant the highest and lowest values discarded (S1 Fig). Results were expressed as area occupied by thrombi in pixel^2^.

**Genotyping**

Screening of CFH, MCP, CFI, CFB, C3 and THBD coding sequences was performed by amplicon-based next generation sequencing (NGS) [6]. Rare functional variants (missense, nonsense, indel, or splicing variants with minor allele frequency, MAF <0.001 in 1000 Genomes and ExAC databases) were selected. Rare variants were defined as likely to be pathogenic, when they were previously associated to complement-related diseases or published functional studies were available, otherwise they were classified as variants of unknown significance. The presence of two SNPs in CFH (rs3753394, -332C>T and rs1065489, p.E936D) that tag the H3 CFH haplotype was also evaluated [7]. The search for genomic abnormalities affecting CFH and CFHR1-5 genes was undertaken using multiplex ligation-dependent probe amplification [8].

**References**

1. World Health Organization. Coronavirus disease (COVID-19) outbreak. Interim guidance ([https://www.who.int/publications-detail/laboratory-testing-for-2019-novel-coronavirus-in suspected-human-cases-20200117](https://www.who.int/publications-detail/laboratory-testing-for-2019-novel-coronavirus-in%20suspected-human-cases-20200117)).

2. Corman VM, Landt O, Kaiser M, Molenkamp R, Meijer A, Chu DK, et al. Detection of 2019 novel coronavirus (2019-nCoV) by real-time RT-PCR. Euro Surveill. 2020;25. doi:10.2807/1560-7917.ES.2020.25.3.2000045

3. Noris M, Galbusera M, Gastoldi S, Macor P, Banterla F, Bresin E, et al. Dynamics of complement activation in aHUS and how to monitor eculizumab therapy. Blood. 2014;124: 1715–1726. doi:10.1182/blood-2014-02-558296

4. Galbusera M, Noris M, Gastoldi S, Bresin E, Mele C, Breno M, et al. An Ex Vivo Test of Complement Activation on Endothelium for Individualized Eculizumab Therapy in Hemolytic Uremic Syndrome. Am J Kidney Dis. 2019;74: 56–72. doi:10.1053/j.ajkd.2018.11.012

5. Bettoni S, Galbusera M, Gastoldi S, Donadelli R, Tentori C, Spartà G, et al. Interaction between Multimeric von Willebrand Factor and Complement: A Fresh Look to the Pathophysiology of Microvascular Thrombosis. J Immunol. 2017;199: 1021–1040. doi:10.4049/jimmunol.1601121

6. Iatropoulos P, Noris M, Mele C, Piras R, Valoti E, Bresin E, et al. Complement gene variants determine the risk of immunoglobulin-associated MPGN and C3 glomerulopathy and predict long-term renal outcome. Mol Immunol. 2016;71: 131–142. doi:10.1016/j.molimm.2016.01.010

7. Caprioli J, Castelletti F, Bucchioni S, Bettinaglio P, Bresin E, Pianetti G, et al. Complement factor H mutations and gene polymorphisms in haemolytic uraemic syndrome: the C-257T, the A2089G and the G2881T polymorphisms are strongly associated with the disease. Hum Mol Genet. 2003;12: 3385–3395. doi:10.1093/hmg/ddg363

8. Valoti E, Alberti M, Iatropoulos P, Piras R, Mele C, Breno M, et al. Rare Functional Variants in Complement Genes and Anti-FH Autoantibodies-Associated aHUS. Front Immunol. 2019;10: 853. doi:10.3389/fimmu.2019.00853
