# Supplementary material for: Eculizumab in patients with severe coronavirus disease 2019 (COVID-19) requiring continuous positive airway pressure ventilator support: Retrospective cohort study
Source: PLoS One. 2021 Dec 20;16(12):e0261113. doi: 10.1371/journal.pone.0261113 (PMC8687582; doi:10.1371/journal.pone.0261113)
Supplement: S2 Appendix — (PDF) [file pone.0261113.s005.pdf]

C5 BLOCKADE WITH ECULIZUMAB (SOLIRIS) THERAPY IN CONTINUOUS POSITIVE AIRWAY PRESSURE (CPAP) VENTILATED PATIENTS WITH CORONAVIRUS DISEASE 2019 (COVID-19)

RETROSPECTIVE OBSERVATIONAL STUDY

Running Title:

Eculizumab to Stop Complement Mediated Lung Damage In Covid 19 Infected Patients

Acronym: SOLID

Final Version: 1.1, January 7th, 2021

Keywords: COVID, eculizumab, CPAP

Prepared by

Piero Ruggenenti*°, Fabiano Di Marco^, Marina Noris°, and Giuseppe Remuzzi*°

*Units of Nephrology and ^Pneumology, ASST Papa Giovanni XXIII

°Istituto di Ricerche Farmacologiche Mario Negri IRCCS

Confidentiality Statement

This protocol contains strictly confidential information which is not to be communicated or published unless previously authorized by sponsor

Approved and signed by:

Prof. Piero Ruggenenti

Unità Operativa Complessa di Nefrologia e Dialisi
ASST Papa Giovanni XXIII
Principal Investigator

Study organization

**Sponsor:**	ASST Papa Giovanni XXIII<br>Piazza OMS, 1, 24127 Bergamo BG<br><br>Istituto di Ricerche Farmacologiche Mario Negri - IRCCS<br>Clinical Research Center for Rare Diseases *Aldo e Cele Daccò*<br>Via G.B. Camozzi, 3, 24020 Ranica, BG
**Clinical Centre:**	ASST Papa Giovanni XXIII
**Coordinating Center:**	Clinical Research Center for Rare Diseases *Aldo e Cele Daccò*<br>Via G.B. Camozzi, 3<br>24020 Ranica, Bergamo, Italy<br>Phone: +39 03545351<br>Fax: +39 0354535371
**Study Statistics:**	Laboratory of Biostatistics<br>Clinical Research Center for Rare Diseases *Aldo e Cele Daccò*<br>*Dr. Annalisa Perna*
**Contract Research Organisation/Monitoring:**	CRO Istituto Mario Negri<br>Laboratory of Pharmacovigilance and Monitoring for Clinical Investigations<br>Laboratory for Regulatory Affairs for Clinical Studies<br>Clinical Research Center for Rare Diseases *Aldo e Cele Daccò*
**eCRF provider:**	Unit of Clinical Research Informatics<br>Department of Biomedical Engineering<br>Clinical Research Center for Rare Diseases *Aldo e Cele Daccò*<br>*Sergio Carminati*

Table of Contents

1. Historical Version 6
2. Background and scientific rationale 7
3. Aim 7
 Primary aim 7
 Safety aims 8
4. Study population 9
 Patients inclusion criteria 9
 Patients exclusion criteria 9
5. Study design 10
6. Considered outcome variables 11
 Primary outcomes 11
 Secondary outcomes 11
 Safety outcomes 12
7. Statistical Analysis 12
8. Centers 12
 Data Management 12
9. Data retention 13
10. Security 13
11. Data Protection 13
12. Study Monitoring 13
 Data Monitoring 13
 Site Audit and Inspections 14
13. Pharmacovigilance 14
14. Ethical, regulatory and administrative aspects 14
 Investigator Responsibilities 14
 Regulatory requirements: Health Authorities and Ethics Committee 14
 Participant confidentiality 14
 Written Informed Consent 15
 Insurance policy 15
 Direct access to original documents 15
 Good Clinical Practice 15
 Essential Document Retention 15
15. References 16

1. Historical Version

Prepared by	Date	Version	List of changes
Piero Ruggenenti<br>Fabiano Di Marco<br>Marina Noris<br>Giuseppe Remuzzi	20.11.2020	1.0	Initial Version
Piero Ruggenenti<br>Fabiano Di Marco<br>Marina Noris<br>Giuseppe Remuzzi	07.01.2021	1.1	Version revised according CE requests

2. Background and scientific rationale

COVID-19

The outbreak of severe acute respiratory syndrome coronavirus 2 (SARS-CoV-2), which originated in Wuhan, China, has become a major concern all over the world. The pneumonia induced by the SARS-CoV-2 is named coronavirus disease 2019 (COVID-19). By October 2020, this virus has affected more than 40 milion people worldwide and caused more than one million of deaths. To date, no specific treatment has been proven to be effective for SARS-CoV-2 infection.

SARS-CoV infection emerged in 2002 to 2003 and led to a global outbreak of SARS. In experimental SARS-CoV the complement cascade in the lung is strongly activated as early as day 1 following SARS-CoV infection. Relative to control mice, SARS-CoV-infected C3 deficient ($C3^{-/-}$) mice exhibited significantly less weight loss, respiratory dysfunction and lung pathology with lower cytokine and chemokine levels despite equivalent viral loads[1]. Consistently, an antihuman C5a antibody similar to eculizumab (IFX-1) remarkably reduced acute lung injury and systemic inflammation in monkeys with severe influenza A (H7N9) pneumonia[2] and OmCI, a potent arthropod-derived inhibitor of C5 activation, significantly inhibited neutrophil and macrophage infiltration in the airways and reduced lung damage induced by Influenza A infection in mice[3].

Eculizumab

Altogether, these findings converge to indicate that inhibition of the terminal complement pathway through pharmacological C5 blockade might be an effective treatment option for patients with severe coronavirus infection, namely those with SARS-CoV-2 induced pneumonia (COVID-19). Eculizumab is a humanized anti-C5 monoclonal antibody approved for the treatment of paroxysmal nocturnal hemoglobinuria[4] and atypical hemolytic uremic syndrome[5]. In these clinical conditions eculizumab is extremely effective, well tolerated and safe. This drug might therefore benefit and even be life-saving in patients with SARS-CoV-2 induced pneumonia (COVID-19). With this background FDA approved a program (ClinicalTrials.gov Identifier: NCT04288713) of off-label compassionate use of the anti C5 monoclonal antibody eculizumab for the treatment of non-intubated patients with COVID-19 [Eculizumab (Soliris) in Covid-19 Infected Patients (SOLID-C19)]. In this context this compassionate use protocol of eculizumab was extended to COVID-19 patients with pneumonia on continuos Positive Airway Pressure (C-PAP) ventilator support at Papa Giovanni XXIII Hospital of Bergamo, Italy.

3. Aim

Primary aim

To evaluate absolute change in respiratory rate from baseline (Visit 1, day 1) to the end of the observation period (Visit 4, day 9).

<u>*Secondary aims*</u>

To evaluate the absolute changes from baseline (Visit 1, day 1) to the end of the observation period (Visit 4 , day 9) in:

- Ex vivo serum-induced complement deposition on resting and ADP-activated human endothelial cells
- Ex-vivo thrombus formation on ADP-activated endothelial cells
- Markers of complement activation in serum and plasma (C3, C5a, C3a, sC5b-9)

To evaluate the absolute changes from baseline (Visit 1, day 1) to the end of the observation period (Visit 4, day 9) in the following continuous outcomes variables including:

- P/F (PaO_2/FiO_2) and $PaCO_2$ from baseline
- Systolic, diastolic and mean blood pressure, respiratory rate and heart rate
- Markers of inflammation or pro-thrombotic milieu including differential WBC count, platelet count, PCR, D-dimer levels

To evaluate the absolute changes from baseline (Visit 1, day 1) to the end of the follow-up period (Visit 6, month 1) in the following continuous outcomes variables including:

- Respiratory rate (breaths/min)
- P/F (PaO_2/FiO_2) and $PaCO_2$ from baseline
- Systolic, diastolic and mean blood pressure, respiratory rate and heart rate
- Markers of inflammation or pro-thrombotic milieu including differential WBC count, platelet count, PCR, D-dimer levels

To evaluate the following end ponts to the end of the extension follow-up period (Visit 9, month 4):

- Time to C-PAP independency from the first eculizumab treatment
- Need of intubation
- Rare and common variants in complement gene
- Discharge without COVID -19 chronic complications
- Death during hospitalization
- Death for all causes
- Death or discharge with COVID-19 chronic complications

<u>*Safety aims*</u>

To evaluate incidence of bacterial or other opportunistic infection and any other clinically relevant event.

4. Study population

Patients and reference-patients were adult (more than 18 years old) subjects with laboratory-confirmed SARS-CoV-2 infection who were admitted at the Pneumology Unit of the Azienda Socio Sanitaria Territoriale (ASST) Papa Giovanni XXIII in Bergamo (Italy) from March 24th, 2020 to April 4th, 2020 because of severe respiratory distress due to SARS-Cov2 induced pneumonia (COVID 19). All of them were on CPAP ventilator support because of severe respiratory insufficiency from 24 hours or less. The diagnosis of COVID-19 was based on the 19 March 2020 WHO Interim guidance criteria[6,] including history of exposure to the virus, typical symptoms and clinical, radiological and laboratory findings upon admission. Radiologic assessments and all laboratory tests, including arterial blood gas analysis, were performed according to local clinical practice and based on clinical needs. The diagnosis was confirmed by detection at admission of SARS-CoV-2 genome from nasal swabs and respiratory samples by using two different molecular methods (GeneFinder COVID-19-Elitech Group, Allplex™ 2019-nCoV Assay - Seegene Inc) according to the manufacturer's instructions. After the purification of viral RNA from clinical samples, the detection of RdRp, E and N viral genes was obtained by real time Polymerase Chain Reaction (RT-PCR) according to WHO protocol.[6,7]

PATIENTS TREATED WITH ECULIZUMAB

Patients received eculizumab treatment according to the *"Eculizumab (Soliris) in Covid-19 Infected Patients (SOLID-C19)"* protocol guidelines in the context of the FDA approved program of off-label compassionate use of the anti C5 monoclonal antibody for the treatment of non-intubated patients with COVID-19 (ClinicalTrials.gov Identifier: NCT04288713). The drug was freely supplied by the manufacturer (Alexion Pharma Italy S.R.L., Milan). Before the first eculizumab administration patients received continued antibiotic coverage against Neisseria Meningitis that was stopped two weeks after the administration of both the seronegative and quadrivalent meningococcal vaccines. The entire vaccination protocol was completed in all participants. All patients provided written informed consent to eculizumab compassionate treatment. None of them received compensation for eculizumab therapy. The compassionate treatment protocol was approved by the local Ethical Committee.

Patients inclusion criteria

- >18 years of age
- COVID-19 pneumonia diagnosed by standard criteria
- Need of Continuous Positive Airway Pressure (CPAP) ventilator support
- Patient written informed consent for compassionate use

Patients exclusion criteria

- Intubation

<u>REFERENCE PATIENTS</u>

As for eculizumab-treated patients, all reference-patients were admitted at the same Pneumology Unit and required CPAP ventilator support because of severe respiratory insufficiency. The diagnosis of COVID-19 was based on the same WHO criteria considered to establish the diagnosis in the ten contemporary patients[6] and, again, was confirmed by detection at admission of SARS-CoV-2 genome from nasal swabs and respiratory samples**.**[6,7] As per center practice, all patients and reference-patients were monitored and treated by the same equip according to the same standardized protocols during the same observation period with the unique exception of eculizumab that was not administered to reference-patients.

5. Study design

Data will be collected at ASST-Papa Giovanni XXIII Hospital of Bergamo. Patient history, physical examinations, vital signs, usual clinical laboratory safety parameters and adverse events recorded in the patient's medical record will be collected according to the standard of care. Ex vivo serum-induced complement deposition on resting and ADP-activated human endothelial cells[8], ex-vivo thrombus formation on ADP-activated endothelial cells,[9] markers of complement activation in plasma (C5a, C3a, sC5b-9)[8] and genetic analysis performed at the Laboratories of the Istituto di Ricerche Farmacologiche Mario Negri IRCCS as per compassionate use protocol will be part of the data collected for this study.

The data recorded in the patient's medical record will be collected at the following timepoints:

	**V1** *Pre infusion*	**V2** *Post infusion*	**V3** *Pre infusion*	**V4** *Post infusion*	**V5** *FU*	**V6** *FU*	**V7** *FU*	**V8** *FU*	**V9** *FU*
	D1	*D2*	*D8*	*D9*	*D15*	*M1*	*M2*	*M3*	*M 4*
Baseline data[1]	X								
Inclusion criteria/Exclusion criteria	X								
Vital signs[2]	X	X	X	X	X	X			
Respiratory assistance assessment[3]	X	X	X	X	X	X			
Blood laboratory examinations[4]	X	X	X	X	X	X			
Biomarkers and viral titer[5]	X	X	X	X	X				
Treatment with Eculizumab[6]	X		X						
Previous and Concomitant Diseases[7]	X								
Concomitant COVID-19 treatments[8]	X								
Concomitant Treatments[9]	X								
Clinical Course[10]	X								

1. Baseline data: patient data, life style, weight and height, hospital admission date, days fo NIV/CPAP before enrollment, symptoms
2. Vital signs: respiratory rate, heart rate, blood pressure, temperature, chest RX/CT for interstitial pneumonia and Barotrauma/PTE
3. Respiratory assistance assessment: pH, pO2, pCO2, HCO3 act, P/F, FiO2, Support, PEEP, Tidal Volume, Pplat, Driving Pressure.
4. Blood laboratory examinations: WBC, ANC, ALC, AMC, PLTs, hemoglobin, CRP, PCT, LDH, AST, ALT, creatinine, D-dimer
5. Biomarkers and viral titer: markers of microangiopathy (fragmented erythrocytes, serum LDH, platelet count, haptoglobin and complete blood cell counts), ex vivo serum-induced complement deposition on resting and ADP-activated human endothelial cells , ex-vivo thrombus formation on ADP-activated endothelial cells, and markers of complement activation in plasma (C5a, C3a, sC5b-9) to evaluate activity of the microangiopathic process and of the complement system up to discharge from the hospital.
6. Eculizumab therapy performed at day 1 and 8 after evaluation pre-infusion
7. Previous and Concomitant Diseases: complete the eCRF with all the events occurred previous and during the hospitalization
8. Concomitant Covid-19 treatments: Ceftriaxone, azithromycin, antiretroviral therapy, methylprednisolone, LMWH, Tocilizumab, hydroxychlorotiazide

9. Concomitant treatments: complete the eCRF with all the concomitant medications/treatment prescribed during the study period
10. Clinical Course: Intubation, date of intubation, ECMO, date of weaning from ECMO, weaning from CPAP, date of weaning from CPAP, length of CPAP treatment, outcome, date of outcome

6. Considered outcome variables

Primary outcomes

Absolute change from baseline (Visit 1, day 1) to the end of the observation period (Visit 4, day 9)

Secondary outcomes

Absolute changes from baseline (Visit 1, day 1) to the end of the observation period (Visit 4 , day 9) in:

- Ex vivo serum-induced complement deposition on resting and ADP-activated human endothelial cells
- Ex-vivo thrombus formation on ADP-activated endothelial cells
- C3, C5a, C3a, sC5b-9
- P/F (PaO2/FiO2) and PaCO2 from baseline
- Systolic, diastolic and mean blood pressure, respiratory rate and heart rate
- Markers of inflammation or pro-thrombotic milieu including differential WBC count, platelet count, PCR, D-dimer levels

Absolute changes from baseline (Visit 1, day 1) to the end of the follow-up period (Visit 6, month 1) in the following continuous outcomes variables including:

- Respiratory rate (breaths/min)
- P/F (PaO2/FiO2) and PaCO2 from baseline
- Systolic, diastolic and mean blood pressure, respiratory rate and heart rate
- Markers of inflammation or pro-thrombotic milieu including differential WBC count, platelet count, PCR, D-dimer levels

The following end points to the end of the extension follow-up period (Visit 9, month 4):

- Time to C-PAP independency from the first eculizumab treatment
- Need of intubation
- Rare and common variants in complement gene;
- Discharge without COVID -19 chronic complications
- Death during hospitalization
- Death for all causes
- Death or discharge with COVID-19 chronic complications

Safety outcomes

- Number of bacterial or other opportunistic infection
- Number of any intercurrent side effect

7. Statistical Analysis

Due to the retrospective and observational nature of the study the sample size has not be calculated. It is expected that patients and reference-patients will be sufficiently comparable at baseline (Visit 1, day 1). However in case of important baseline imbalances a matching will be attempted between the two groups. Control patients will be matched with the ten treated patients by using the "Propensity Score" SAS procedure among the other patients, affected by COVID-19, from the units of Nephrology and Pneumology of ASST Papa Giovanni XXIII. Scores will be built with a logistic regression, which will consider age, gender and the main baseline charateristics of patients, in order to obtain a matching of two controls for each treated patient.
Continuous variables will be analyzed through descriptive statistics and reported as mean (SD) or median [IQR], as appropriate. Within-group changes with respect to baseline will be analyzed with paired T- test or signed- rank Wilcoxon test, as appropriate. Differences between groups will be analysed by analysis of covariance (ANCOVA) or chi-square or Fisher's exact test in case of continuous or categorical data, as appropriate. Survival analysis will be performed by means of Cox proportional hazard regression models and results will be expressed as hazard ratio (HR) and 95% confidence interval (CI). Cumulative events will be constructed using he Kaplan-Meier method. Statistically significant differences will be assumed at 5% level of probability.

8. Centers

This is a single center retrospective observational study. All data will be collected at the COVID Units of ASST Papa Giovanni XXIII, Bergamo, Italy.

Data Management

All study data will be collected by the Investigator and/or other study personnel. A clinical trial database will be provided, in which the data are entered via an electronic Case Report Form (eCRF). Authorized and trained staff of the study center will enter the data in the eCRF.
For each participant enrolled, an eCRF must be completed after the participant's visit. This also applies to records for those participants who fail to complete the study.
The Investigator should ensure the accuracy, completeness and timelines of the data reported in the eCRFs and all required reports.
Verification of the data in the eCRF will occur by monitoring as well as by range, validity, and consistency checks programmed in the system. In certain cases, queries can be detected by the study software or by

authorized study staff. Based on the queries, the Investigator can review and answer the found discrepancies directly in the system. All changes of data entered in the eCRF can be followed by an audit trial.
Quality control will be performed before the database is closed. This procedure will be documented. Finally, data transfer will take place for statistical evaluation.

9. Data retention

We will pseudo-anonymize (de-identify) rather than permanently (and irreversibly) anonymize data in the data retention process. Depersonalization means that data with identifying information is collected, but the identifying information is then severed from the personal health information data in the research database and is stored separately. Identifying information linking to the research database will be kept at the local site. No identifying information will be stored in an electronic form.

10. Security

Research data transferred between all local sites and the central data repository will been crypted. This will be realized with the help of HTTPS (HTTP over SSL). To prevent unauthorized access to the Research Database, each user has to login with his personal user name and password. Passwords have to be changed in a regular manner and have to meet a defined complexity (minimum length, mix of characters, numbers and special characters).

11. Data Protection

All study staff will give due consideration to data protection and medical confidentiality. The collection, transfer, storage and analysis of personal study-related data are performed pseudo-anonymized according to national regulations.

12. Study Monitoring

Data Monitoring

The study will be monitored by the staff of the Laboratory of Pharmacovigilance and Monitoring for Clinical Investigations (Istituto di Ricerche Farmacologiche Mario Negri - IRCCS). If requested, the investigator agrees to allow monitoring visits/audits on site prior, during and after the completion of the study.
This study will make use of a computerized system that allows data capture, monitoring and storage of clinical data. The system eCRF fulfils all the formal requirements of the Good Clinical Practice (GCP) and FDA guidance in order to assure adequate standards of data quality, safety and protection, in the absence of paper CRF.

Site Audit and Inspections

Audits (by the Sponsor) and inspections (by regulatory authorities) may be performed in order to verify that the clinical study is performed according to the study protocol as well as to other applicable regulatory requirements. The auditor or inspector is independent in regard to personnel involved in the conduct of this clinical trial. This may occur at any time from start to after closure of the study.

13. Pharmacovigilance

This is a an observational study so any adverse reaction recorded during the patient follow up must be reported by the investigator according the regulations in force for spontaneous post-marketing reports.

14. Ethical, regulatory and administrative aspects

Investigator Responsibilities

Investigator responsibilities are set out in ICH-GCP.

The investigators will conduct the study in compliance with the protocol given approval/favourable opinion by the ethics committee and the national competent bodies.

Investigators must enter study data in e-CRF within 1 week after the visit. The investigator will permit study-related monitoring visits and audits by the Sponsor or its representatives as well as regulatory inspection(s). The investigator must provide direct access to the study center's facilities, to source documents, and to all other study documents.

The investigator or a designated member of the investigator's staff must be available during monitoring visits to review data and resolve any queries and to allow direct access to the participant's records (e.g., medical records, office charts, hospital charts, and study related charts) for source data verification. The data collection must be completed prior to each monitoring visit so that the accuracy and completeness may be checked.

Regulatory requirements: Health Authorities and Ethics Committee

The study protocol will be conducted in accordance with ICH-GCP requirements, national laws and the declaration of Helsinki and will be approved by the institutional review board.

The study protocol and all the other appropriate documents will be submitted to the Independent Ethic Committee (IEC), in accordance with local legal requirements.

Participant confidentiality

In order to maintain confidentiality, all eCRFs, study reports and communications regarding the study will identify the study participants by the assigned unique trial identifier only. Participant confidentiality will be maintained at every stage; participant data will not be made publicly available to the extent permitted by the applicable laws and regulations.

Written Informed Consent

Doctor must inform patient and caregiver of the aims of the study and how it will be organized, the type of study analyses, the anticipated benefits which can be expected from the study, any potential hazards of the study and discomfort it may entail, the freedom to ask for further information at any time, the patient's right to withdraw from the study at any time without giving reasons, the existence, if applicable, of patient's insurance cover and obligations following from this cover.

No patient should be obliged to participate in the trial. The patient must be given ample opportunity to enquire about details of the study. If there is any doubt as to whether the patient has understood the written and verbal information, the patient should not enter the study.

Insurance policy

This is a retrospective observational study, study insurance not required.

Direct access to original documents

The Investigator/Institution shall allow Regulatory Authorities, national and foreign, and personnel designated by the Independent Ethical Committee or the Sponsor, direct access and related verification to all study original documentation including forms of informed consent signed by the patients' part of the study and clinical and/or outpatient records. Those having direct access to such documentation shall take the necessary reasonable precautions in order to keep identity of patients and information owned by the Sponsor confidential, in accordance with the applicable regulating requirement.

Good Clinical Practice

The trial will be carried out in accordance with the principles of the ICH-GCP guidelines (www.ich.org). All Investigators and member of Research Staff will be GCP trained and this training has to be certified.

Essential Document Retention

The investigator will retain copies of all the essential documents as required by the applicable regulatory requirements. The investigator should take measures to prevent accidental or premature destruction of these documents.

The essential documents include at least: the signed protocol, copies of the completed CRFs, hospital records, and other source documents, IEC submission and all related correspondence, and all other documentation included in the investigator site file.

15. References

1. Gralinski LE, Sheahan TP, Morrison TE, Menachery VD, Jensen K, Leist SR, Whitmore A, Heise MT, Baric RS. Complement Activation Contributes to Severe Acute Respiratory Syndrome Coronavirus Pathogenesis. mBio. 2018 Oct 9;9(5). pii: e01753-18. doi: 10.1128/mBio.01753-18.
2. Sun S, Zhao G, Liu C, Fan W, Zhou X, Zeng L, Guo Y, Kou Z, Yu H, Li J, Wang R, Li Y, Schneider C, Habel M, Riedemann NC, Du L, Jiang S, Guo R, Zhou Y. Treatment with anti-C5a antibody improves the outcome of H7N9 virus infection in African green monkeys. Clin Infect Dis. 2015 Feb 15;60(4):586-95. doi: 10.1093/cid/ciu887. Epub 2014 Nov 27.
3. Garcia CC, Weston-Davies W, Russo RC, Tavares LP, Rachid MA, Alves-Filho JC, Machado AV, Ryffel B, Nunn MA, Teixeira MM. Complement C5 activation during influenza A infection in mice contributes to neutrophil recruitment and lung injury. PLoS One. 2013 May 16;8(5):e64443. doi: 10.1371/journal.pone.0064443. Print 2013.
4. Hillmen P, Young NS, Schubert J, et al. The complement inhibitor eculizumab in paroxysmal nocturnal hemoglobinuria. N Engl J Med. 2006;355(12): 1233-1243.
5. Gruppo RA, Rother RP. Eculizumab for congenital atypical hemolytic-uremic syndrome. N Engl J Med. 2009;360(5): 544-546.
6. World Health Organization. Coronavirus disease (COVID-19) outbreak. Interim guidance (https://www.who.int/publications-detail/laboratory-testing-for-2019-novel-coronavirus-in suspected-human-cases-20200117).
7. Corman VM, Landt O, Kaiser M, et al. Detection of 2019 novel coronavirus (2019-nCoV) by real-time RT-PCR. Euro surveillance: bulletin Europeen sur les maladies transmissibles = European communicable disease bulletin 2020; 25(3).
8. Noris M, Galbusera M, Gastoldi S, Macor P, Banterla F, Bresin E, Tripodo C, Bettoni S, Donadelli R, Valoti E, Tedesco F, Amore A, Coppo R, Ruggenenti P, Gotti E, Remuzzi G. Dynamics of complement activation in aHUS and how to monitor eculizumab therapy. Blood. 2014 Sep 11;124(11):1715-26. doi: 10.1182/blood-2014-02-558296. Epub 2014 Jul
9. Bettoni S, Galbusera M, Gastoldi S, Donadelli R, Tentori C, Spartà G, Bresin E, Mele C, Alberti M, Tortajada A, Yebenes H, Remuzzi G, Noris M.. J Immunol. 2017 Aug 1;199(3):1021-1040.
