# Supplementary material for: Eculizumab in patients with severe coronavirus disease 2019 (COVID-19) requiring continuous positive airway pressure ventilator support: Retrospective cohort study
Source: PLoS One. 2021 Dec 20;16(12):e0261113. doi: 10.1371/journal.pone.0261113 (PMC8687582; doi:10.1371/journal.pone.0261113)
Supplement: S3 Appendix — (DOCX) [file pone.0261113.s006.docx]

STROBE Statement—Checklist of items that should be included in reports of ***cohort studies***

|  | Item No | Recommendation | Page No |
| --- | --- | --- | --- |
| **Title and abstract** | 1 | (a) Indicate the study’s design with a commonly used term in the title or the abstract | (title:  “Retrospective cohort study”) |
|  |  | (b) Provide in the abstract an informative and balanced summary of what was done and what was found | Abstract |
| Introduction | | | |
| Background/rationale | 2 | Explain the scientific background and rationale for the investigation being reported | “Introduction” |
| Objectives | 3 | State specific objectives, including any prespecified hypotheses | “Introduction” last sentence “Based on this background..”  Study Protocol |
| Methods | | | |
| Study design | 4 | Present key elements of study design early in the paper | “Materials and Methods” Section:  Headings: “Study population”,  “Eculizumab compassionate treatment”,  “Follow-up”  Study Protocol |
| Setting | 5 | Describe the setting, locations, and relevant dates, including periods of recruitment, exposure, follow-up, and data collection | “Materials and Materials” Section:  Heading “Study population”  Study Protocol |
| Participants | 6 | (a) Give the eligibility criteria, and the sources and methods of selection of participants. Describe methods of follow-up | “Materials and Methods” Section:  Headings “Study population”,  “Eculizumab compassionate treatment”,  “Follow-up” |
|  |  | (b) For matched studies, give matching criteria and number of exposed and unexposed | Study Protocol |
| Variables | 7 | Clearly define all outcomes, exposures, predictors, potential confounders, and effect modifiers. Give diagnostic criteria, if applicable | Heading “Sample Size and Statistical Analyses”  3^st^ paragraph “Primary outcome…”  4^st^ paragraph “Secondary short-term….”  5^st^ paragraph  “The combined endpoint….”  7^st^ paragraph  “Analyses were adjusted by….”  Study Protocol |
| Data sources/ measurement | 8* | For each variable of interest, give sources of data and details of methods of assessment (measurement). Describe comparability of assessment methods if there is more than one group | “Materials and Methods” Section:  Headings “Study population”,  “Eculizumab compassionate treatment”,  “Follow-up”  “Complement evaluation and genotyping” |
| Bias | 9 | Describe any efforts to address potential sources of bias | Heading “Sample Size and Statistical Analyses”  9^st^ sentence  “We reported…”  10^st^ sentence  “Survival analysis…”  Study Protocol |
| “Study size | 10 | Explain how the study size was arrived at | Heading “Sample Size and Statistical Analyses”  2^st^ paragraph  “Due to the retrospective…”  Study Protocol |
| Quantitative variables | 11 | Explain how quantitative variables were handled in the analyses. If applicable, describe which groupings were chosen and why | Heading “Sample Size and Statistical Analyses”  Study Protocol |
| Statistical methods | 12 | (a) Describe all statistical methods, including those used to control for confounding | Heading “Sample Size and Statistical Analyses”  Study Protocol |
|  |  | (b) Describe any methods used to examine subgroups and interactions |  |
|  |  | (c) Explain how missing data were addressed |  |
|  |  | (d) If applicable, explain how loss to follow-up was addressed |  |
|  |  | (e) Describe any sensitivity analyses |  |
| Results | | |  |
| Participants | 13* | (a) Report numbers of individuals at each stage of study—eg numbers potentially eligible, examined for eligibility, confirmed eligible, included in the study, completing follow-up, and analysed | “Results” Section  Heading  “Long term endpoints”  Figure 4 |
|  |  | (b) Give reasons for non-participation at each stage |  |
|  |  | (c) Consider use of a flow diagram |  |
| Descriptive data | 14* | (a) Give characteristics of study participants (eg demographic, clinical, social) and information on exposures and potential confounders | “Results” Section Table 1, Table 2 |
|  |  | (b) Indicate number of participants with missing data for each variable of interest |  |
|  |  | (c) Summarise follow-up time (eg, average and total amount) |  |
| Outcome data | 15* | Report numbers of outcome events or summary measures over time | “Results” Section  Heading  “Long term endpoints”  Figure 4  Tables S1, S2 |

| Main results | 16 | (a) Give unadjusted estimates and, if applicable, confounder-adjusted estimates and their precision (eg, 95% confidence interval). Make clear which confounders were adjusted for and why they were included | “Results” Section  Heading  “Long term endpoints”  Figure 4 |
| --- | --- | --- | --- |
|  |  | (b) Report category boundaries when continuous variables were categorized |  |
|  |  | (c) If relevant, consider translating estimates of relative risk into absolute risk for a meaningful time period |  |
| Other analyses | 17 | Report other analyses done—eg analyses of subgroups and interactions, and sensitivity analyses | “Results” Section  Heading “Complement activity and ex vivo complement deposition and thrombogenesis”  Figures 1-3 |
| Discussion | | | |
| Key results | 18 | Summarise key results with reference to study objectives | “Discussion” Section |
| Limitations | 19 | Discuss limitations of the study, taking into account sources of potential bias or imprecision. Discuss both direction and magnitude of any potential bias | “Discussion” Section  Last paragraph “Major study limitations were…” |
| Interpretation | 20 | Give a cautious overall interpretation of results considering objectives, limitations, multiplicity of analyses, results from similar studies, and other relevant evidence | “Discussion” Section  Last sentence “In conclusion…” |
| Generalisability | 21 | Discuss the generalisability (external validity) of the study results | “Discussion” Section  Last paragraph “Major strengths…” |
| Other information | | | |
| Funding | 22 | Give the source of funding and the role of the funders for the present study and, if applicable, for the original study on which the present article is based | Submission form |

*Give information separately for exposed and unexposed groups.

**Note:** An Explanation and Elaboration article discusses each checklist item and gives methodological background and published examples of transparent reporting. The STROBE checklist is best used in conjunction with this article (freely available on the Web sites of PLoS Medicine at http://www.plosmedicine.org/, Annals of Internal Medicine at http://www.annals.org/, and Epidemiology at http://www.epidem.com/). Information on the STROBE Initiative is available at http://www.strobe-statement.org.
